# Publisher Correction: Mergeable nervous systems for robots

**DOI:** 10.1038/s41467-017-01622-0

**Published:** 2017-11-21

**Authors:** Nithin Mathews, Anders Lyhne Christensen, Rehan O’Grady, Francesco Mondada, Marco Dorigo

**Affiliations:** 1 IRIDIA CoDE, Université Libre de Bruxelles, Brussels, 1050 Belgium; 2 Instituto Universitário de Lisboa (ISCTE-IUL), Instituto de Telecomunicações, Lisbon, 1649-026 Portugal; 3 Institut de Microinformatique (IMT), Faculté des Sciences et Technique de l’Ingénieur (STI), Ecole polytechnique fédérale de Lausanne, Lausanne, 1015 Switzerland

*Nature Communications* **8**:439 doi:10.1038/s41467-017-00109-2; Article published online 12 September 2017

The original version of this Article contained an error in the author contributions section, whereby credit for design of the experiments was not attributed to N.M. This error has now been corrected in both the PDF and HTML versions of the Article.

