# Supplementary material for: Mergeable nervous systems for robots
Source: Nat Commun. 2017 Sep 12;8:439. doi: 10.1038/s41467-017-00109-2 (PMC5595853; doi:10.1038/s41467-017-00109-2)
Supplement: Supplementary file 1 — Supplementary information [file 41467_2017_109_MOESM1_ESM.pdf]

File name: Supplementary Information
Description: Supplementary figures and supplementary table 1

File name: Supplementary Movie 1
Description: **Demonstrating morphological and nervous system flexibility**. MNS robots autonomously merge and split to form bodies of different shapes and sizes.

File name: Supplementary Movie 2
Description: **Morphology and size independent reactions to an external stimulus**. Ten MNS robots react to a stimulus by pointing at it with their green LEDs, and retreating when the stimulus is too close (point-and-retreat behavior). The ten robots then merge to form two MNS robots, which display the same point and retreat behavior. The two MNS robots then merge into a single MNS robot which displays the same point-and-retreat behavior.

File name: Supplementary Movie 3
Description: **Borrowing object lifting capabilities.** An MNS robot extends its morphology to acquire new physical capabilities.

File name: Supplementary Movie 4
Description: **Self-healing**. First segment: an MNS robot recovers from a fault in its brain unit. Second segment: an MNS robot recovers from a fault in one of the robotic units of its body.

File name: Peer Review File
Description:

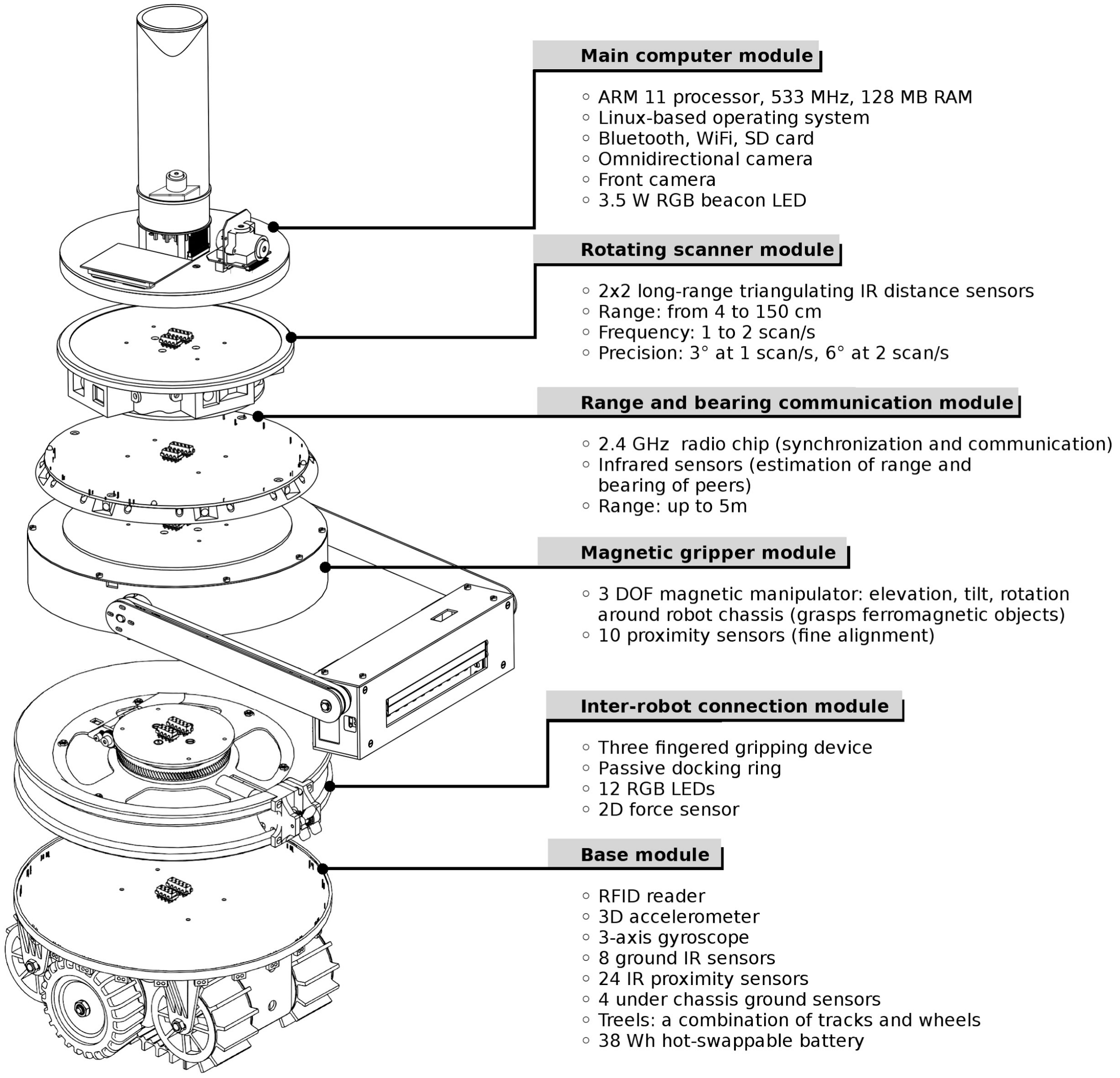

Supplementary Fig. 1: The marXbot robotic platform. In this study, each robotic unit in an MNS robot is a single marXbot robot. The marXbot is a robot whose hardware configuration can be modified through the inclusion and exclusion of particular modules. This design allows us to create MNS robots with different physical abilities.

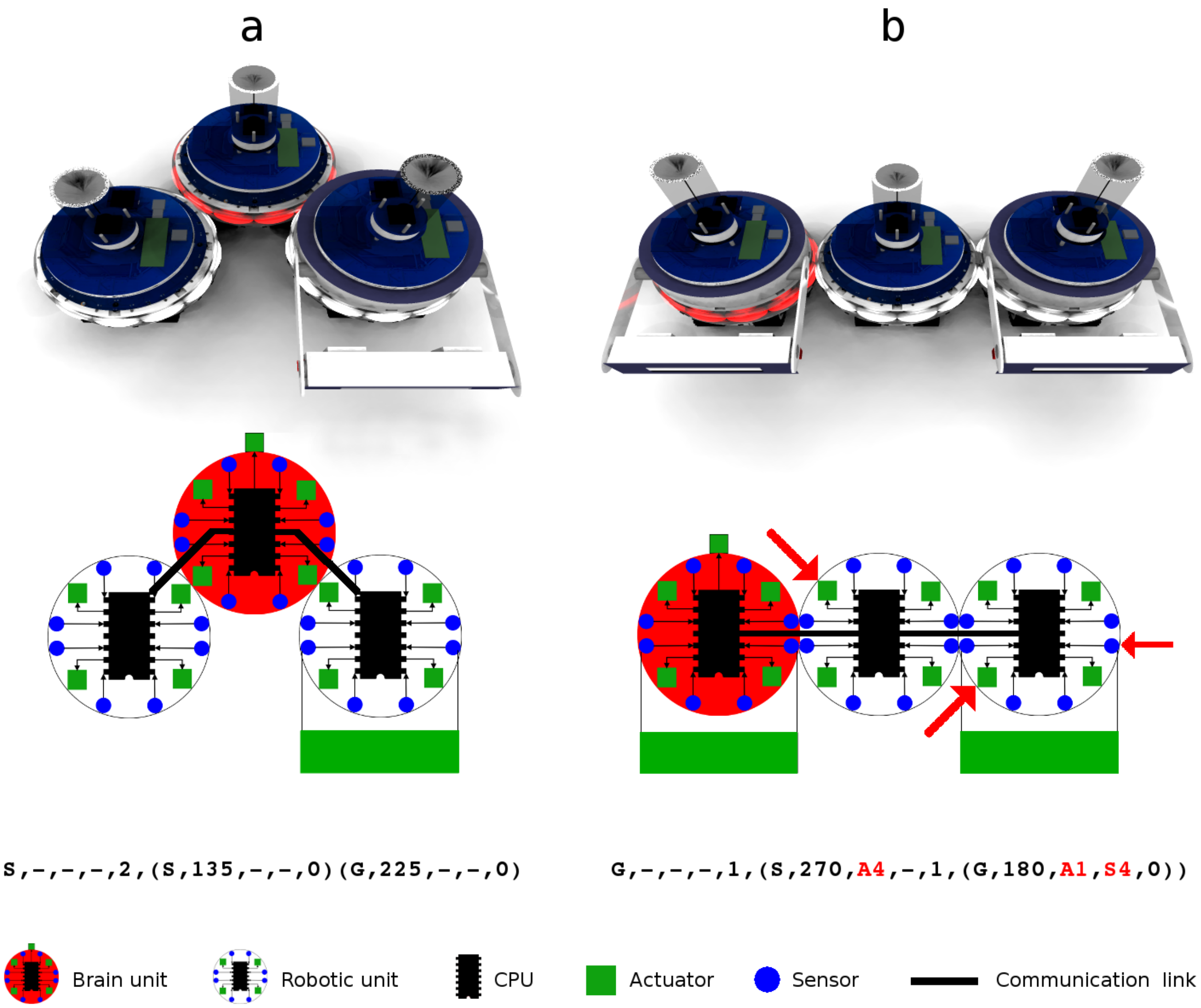

Supplementary Fig. 2: Brain units' internal representation of robot morphology. (**a**,**b**) Two examples of three-unit MNS robots with different morphologies. Two different robotic unit hardware configurations are used, represented for brevity by letters that stand in for the full geometric description. Hardware configuration ‘S’ is a standard marXbot, while hardware configuration ‘G’ is a marXbot where the distance scanner module has been replaced by a magnetic gripper module. The rendering at the top of each pane shows the configuration of robotic units in the robot. The illustration underneath shows the corresponding merged robot nervous system topology. At the bottom of each pane, the serialized version of the brain unit's internal representation is shown (see Methods). The arrows in pane (**b**) indicate malfunctioning sensors and actuators, which are also highlighted in the string serialization beneath.

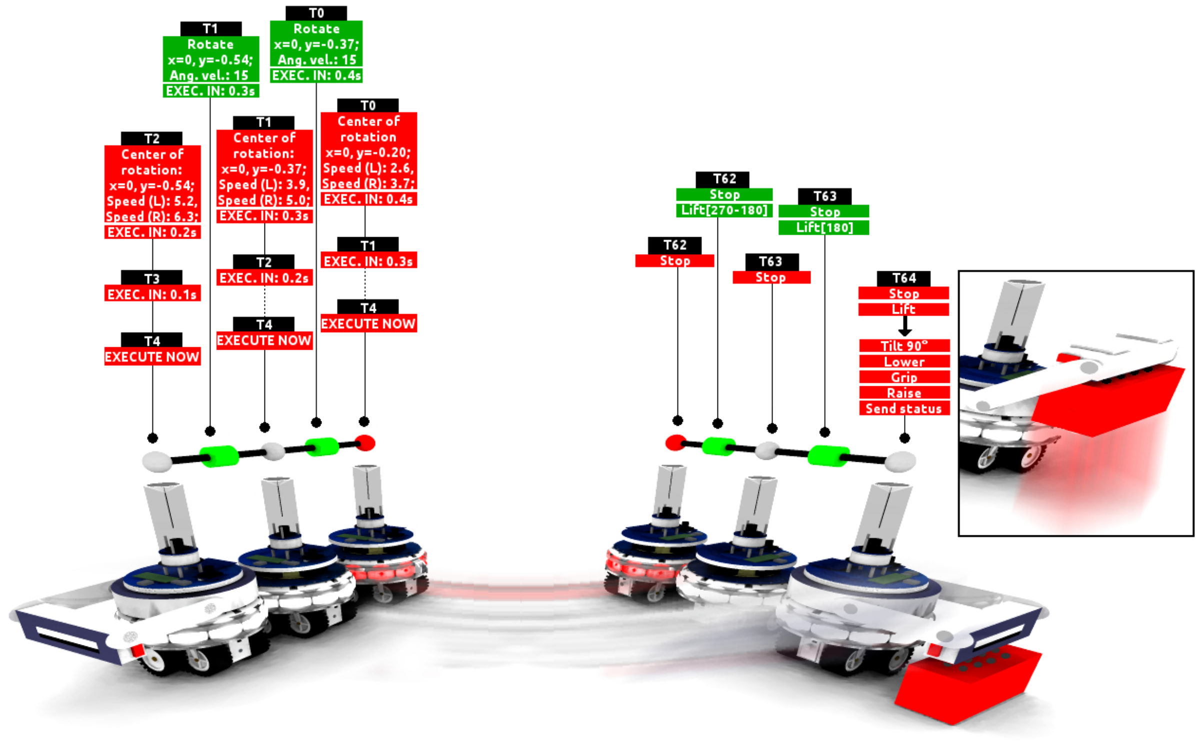

Supplementary Fig. 3: Spatial and temporal actuator coordination in an MNS robot. Black rectangles show timesteps. Each timestep lasts 0.1 seconds, so, for example, T3 occurs 0.3 seconds after timestep T0. Green rectangles contain the high-level control logic being communicated. Red rectangles show what low-level actions each robotic unit is planning, in order to execute the high-level commands received. **Left**: At time T0, the brain unit has detected a red brick it wants to lift using the magnetic gripper module. To access the brick, the MNS robot first needs to rotate. To ensure synchronized movement of its body, the brain unit does not execute the rotation action immediately. Instead, it starts a countdown of 0.4 seconds until execution. In the same timestep (T0), the brain unit sends the high-level `Rotate` command (see Supplementary Table 1 for details) to its child robotic unit – the middle robotic unit. The command contains rotation coordinates in the middle robotic unit's spatial frame of reference. The communication takes a single timestep, so at time T2, the middle robotic unit launches its own countdown (which starts at 0.3 seconds). A similar communication of the high-level `Rotate` command happens between the middle robotic unit and its child robotic unit at timestep T1. At T4, each robotic unit in the MNS robot executes its low-level actuator instruction, and the whole robot rotates in a coordinated fashion. Note that the brain unit is not responsible for controlling individual wheel actuators. It just indicates the center of rotation and the individual robotic units are responsible for determining their local wheel speeds to realize the rotation. **Right**: Once rotated, the brain unit issues a high-level commands to stop (`Stop`) the rotation and to lift (`Lift`) the brick. The brain unit specifies which of the robotic units in its body should execute the lift (the '270-180' in the communication at T62 uniquely identifies the robotic unit with the magnetic gripper module). Once the lift command arrives at the robotic unit with the magnetic gripper module, the robotic unit translates the high-level command into the appropriate sequence of low-level actions. **Inset**: the brick after the lift command has been executed.

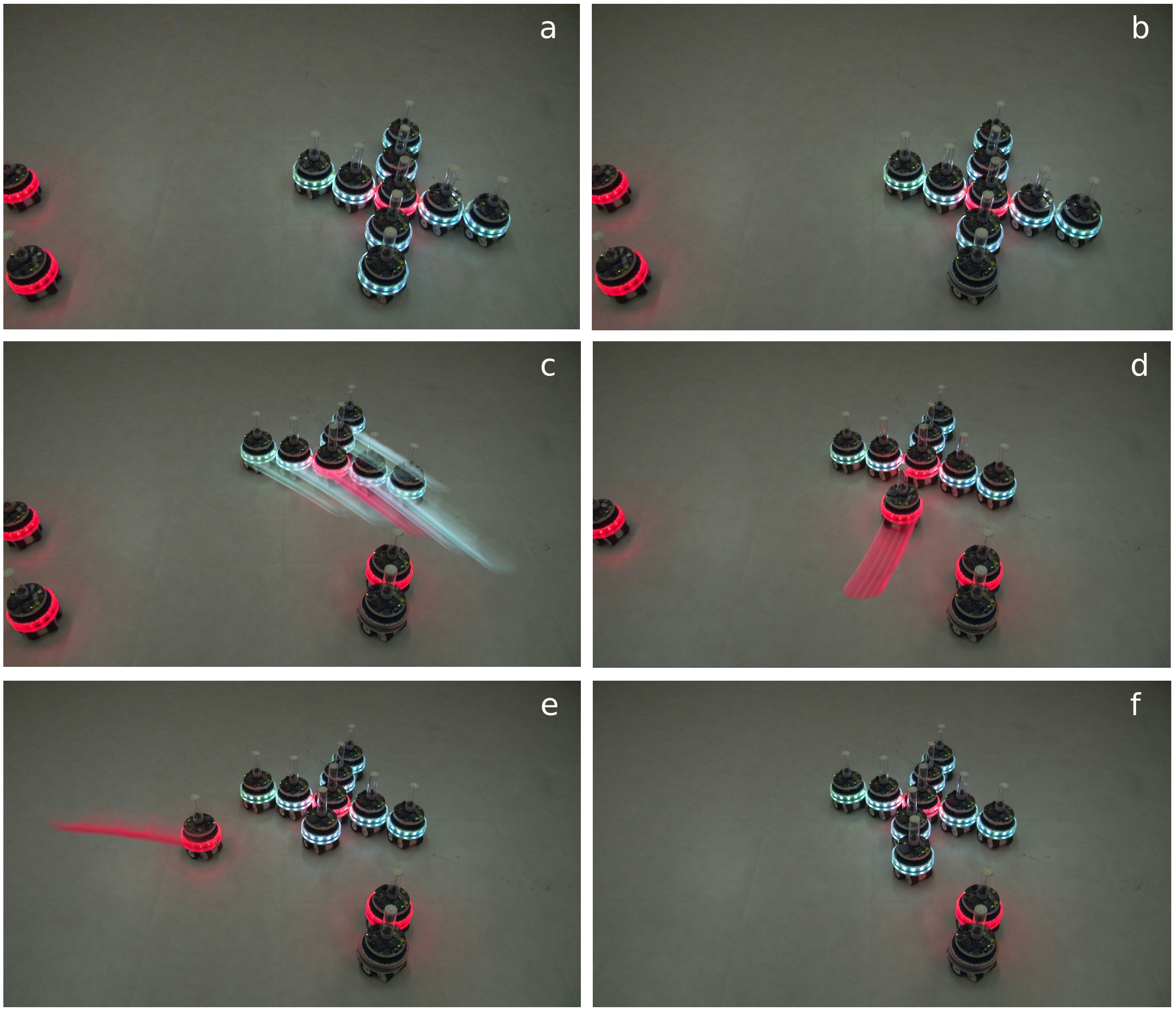

Supplementary Fig. 4: An MNS robot recovers from a fault in a non-brain robotic unit. Photos are snapshots from an experiment (Supplementary Movie 4). (**a**,**b**) A robotic unit fails. Using a heartbeat protocol, the parent robotic unit detects the fault, and communicates this information to the brain unit. (**c**) As the faulty robotic unit is unable to take any action and disconnect from the MNS robot, the brain unit instructs the parent of the faulty robotic unit to sacrifice itself and disconnect. The healthy part of the MNS robot moves away from the immobilized robotic units (faulty robotic unit and its parent). (**d**,**e**,**f**) Free robots are recruited to recreate the original morphology.

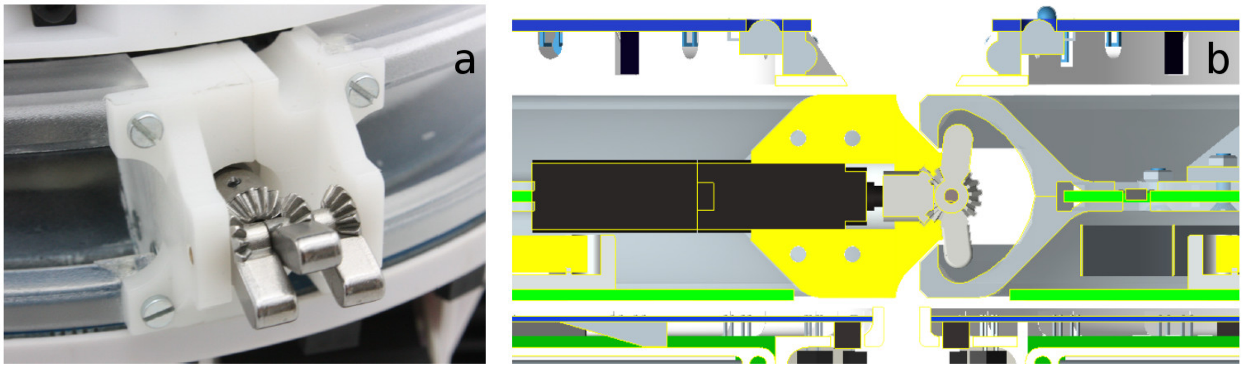

Supplementary Fig. 5: The three-fingered attachment device. (**a**) Close up photo of the three-fingered attachment device. (**b**) A physical connection between marXbots. The attaching marXbot (on the left) has opened its three-fingered attachment device inside the passive docking ring of the marXbot on the right.

**SENSOR / ACTUATOR MESSAGES**	
COMMAND	PAYLOAD
**MESSAGES ADDRESSED TO SPECIFIC UNITS OR GROUPS OF UNITS**	
Extend[*address*]	<attachment angle>;<recruit id>
Disconnect[*address*]	<attachment angle>
SetLedColors[*address*]	<led color 1>; …; <led color 12>
Lift[*address*]	<object bearing>
GetCameraObjects[*address*]	<request type><br>The <request type> parameter for this command and those below is of the form:<br>[once|duration=x;frequency=y|conditional=if_changed]
GetBatteryLevel[*address*]	<request type>
GetAccelerometerReading[*address*]	<request type>
GetProximitySensorReadings[*address*]	<request type>
GetGroundSensorReadings[*address*]	<request type>
Heartbeat[*address*]	<emission frequency>;<reaction to failure=[disconnect_retract|become_brain]>
**MESSAGES ADDRESSED TO ALL UNITS (WHOLE MNS ROBOT)**	
Move	<speed>;<heading>
Rotate	<center of rotation>;<angular velocity>
Stop	(none)

Supplementary Table 1: Sensor and actuator messages. An addressing system allows the brain unit to target these messages at particular robotic units, or sets of robotic units. The header of each message contains the recipient-unit address. The address takes the form of a path from the brain unit down through the MNS robot. The path consists of a sequence of angles corresponding to connections between physically connected robotic units. For example, a message with address header '246-197-180' follows a path to the child robotic unit connected to the brain unit at 246°, then to the next child robotic unit connected to the brain unit's child at 197° (the brain unit's grandchild), and finally to the next child robotic unit connected at 180°. This robotic unit at the end of the path is the intended recipient of the message.
